# Complete Nucleotide Sequences of Virulence-Resistance Plasmids Carried by Emerging Multidrug-Resistant *Salmonella enterica* Serovar Typhimurium Isolated from Cattle in Hokkaido, Japan

**DOI:** 10.1371/journal.pone.0077644

**Published:** 2013-10-14

**Authors:** Yukino Tamamura, Kiyoshi Tanaka, Masato Akiba, Toru Kanno, Shinichi Hatama, Ryoko Ishihara, Ikuo Uchida

**Affiliations:** 1 Hokkaido Research Station, National Institute of Animal Health, Sapporo, Hokkaido, Japan; 2 National Institute of Animal Health, Tsukuba, Ibaraki, Japan; 3 Graduate School of Life and Environmental Sciences, Osaka Prefecture University, Izumisano, Osaka, Japan; 4 Exotic Disease Research Division, National Institute of Animal Health, Kodaira, Tokyo, Japan; 5 United Graduate School of Veterinary Sciences, Gifu University, Yanagido, Gifu, Japan; University of Osnabrueck, Germany

## Abstract

In the present study, we have shown that virulence-resistance plasmids from emerging multidrug-resistant isolates of *Salmonella enterica* serovar Typhimurium were derived from a virulence-associated plasmid, essential for systematic invasiveness of *S*. Typhimurium in mice (pSLT), through acquisition of a large insert containing a resistance island flanked by IS*1294* elements. A *bla*_CMY-2_-carrying plasmid from a cefotaxime-resistant isolate comprised a segment of *Escherichia coli* plasmid pAR060302 and the replication region (IncFIB) of a virulence-resistance plasmid. These results provide insights into the evolution of drug resistance in emerging clones of *S*. Typhimurium.

## Introduction

Salmonella enterica serovar Typhimurium frequently causes salmonellosis in humans and animals. Selection pressure in livestock or humans selects for multidrug-resistant bacterial clones. *S. enterica* epidemics often involve rapid dissemination of the predominant epidemic strains over a large geographic area. For example, the definitive phage type 104 (DT104) emerged during the early 1990s and is spreading through many countries [1–3]. Its resistance to ampicillin, chloramphenicol, streptomycin, sulfonamides, and tetracycline is encoded by the chromosomal locus SGI1, which contains class 1 integrons [4–7]. Concurrently, the incidence of bovine salmonellosis caused by *S*. Typhimurium DT104 increased, particularly in adult cattle in Hokkaido, Japan [8]. In 2002, we identified a non-DT104 multidrug-resistant clone with a pulsed-field gel electrophoresis (PFGE) pattern designated PFGE cluster VII and no recognized phage-type disseminating among cattle in Hokkaido [8]. These isolates carry the *bla*_TEM-1_ gene encoding TEM-1 β-lactamase on *S*. Typhimurium serovar-specific virulence plasmids [8]. The virulence plasmid in *S*. Typhimurium has a highly conserved *spv* locus, which increases its intracellular proliferation in the host during the extraintestinal phase of disease [9,10]. Twenty-five of 165 PFGE cluster VII isolates exhibited *bla*_CMY-2_-mediated cefazolin resistance localized within the chromosomal genomic island GI-VII-6 [11]. One isolate is resistant to the third-generation cephalosporin, cefotaxime, and harbors a virulence-resistance plasmid and a *bla*_CMY-2_-carrying plasmid [8].

To understand the nature of antibiotic resistance in these emerging multidrug-resistant clones, we determined the complete sequences of novel virulence-resistance plasmids and a *bla*_CMY-2_-carrying plasmid from *S*. Typhimurium isolates classified as PFGE cluster VII.

## Materials and Methods

### Plasmids

The virulence-resistance plasmids identified using Southern hybridization with *spvC* and *bla*_TEM-1_ probes were extracted from *S*. Typhimurium KT161 and KT262 that were isolated from diseased cattle in Hokkaido in 2002 and 2005, respectively, and are representative isolates of PFGE cluster VII [8]. The *bla*_CMY-2_-carrying plasmid was also identified using Southern hybridization and extracted from cefotaxime-resistant *S*. Typhimurium TST207 (PFGE cluster VII) isolated from diseased cattle in Hokkaido in 2005 [8].

### Nucleotide sequence analysis

The plasmids were introduced into *Escherichia coli* DH5α (Takara Co. Ltd., Kyoto, Japan) using electroporation (1.25 kV, 100 Ω, 25 μF). The cells were plated on Luria-Bertani agar plates containing 50 μg·ml^−1^ ampicillin or 30 μg·ml^−1^ cefazolin. Plasmid DNA was sequenced by Hokkaido System Science Ltd. (Sapporo, Hokkaido, Japan) using the RocheFLX System (Roche Applied Science, Mannheim, Germany). The GS De Novo Assembler (Roche Applied Science) software was used to assemble sequences. Predicted gaps between contigs were amplified using specific polymerase chain reaction primers and sequenced using the Sanger technique by primer walking. Takara LA Taq polymerase (Takara Co. Ltd., Kyoto, Japan) was used to amplify fragments. Plasmid sequences were annotated using DDBJ Microbial Genome Annotation Pipeline ver. 1.06 (https://migap.lifesciencedb.jp/mgap/jsp/index.jsp). Sequence alignments were generated using BLAST, and plasmid sequences were compared using the Artemis Comparison Tool (ACT) (http://www.sanger.ac.uk/resources/software/act/).

### Nucleotide sequence accession numbers

The annotated nucleotide sequences of pYT1, pYT2, pYT3, and the partial sequence of pYT4 were submitted to the DDBJ database under the accession numbers AB576781, AB605179, AB591424, and AB723628, respectively.

## Results and Discussion

Plasmids pYT1 and pYT2 were isolated from KT161 and KT262, respectively, and plasmids pYT3 and pYT4 were isolated from TST207. The pYT1, pYT2, and pYT4 transformants were resistant to ampicillin, streptomycin, sulfonamide, tetracycline, and kanamycin. The pYT3 transformant was resistant to ampicillin, cefazolin, chloramphenicol, streptomycin, sulfonamides, and tetracycline.

**Figure 1:**
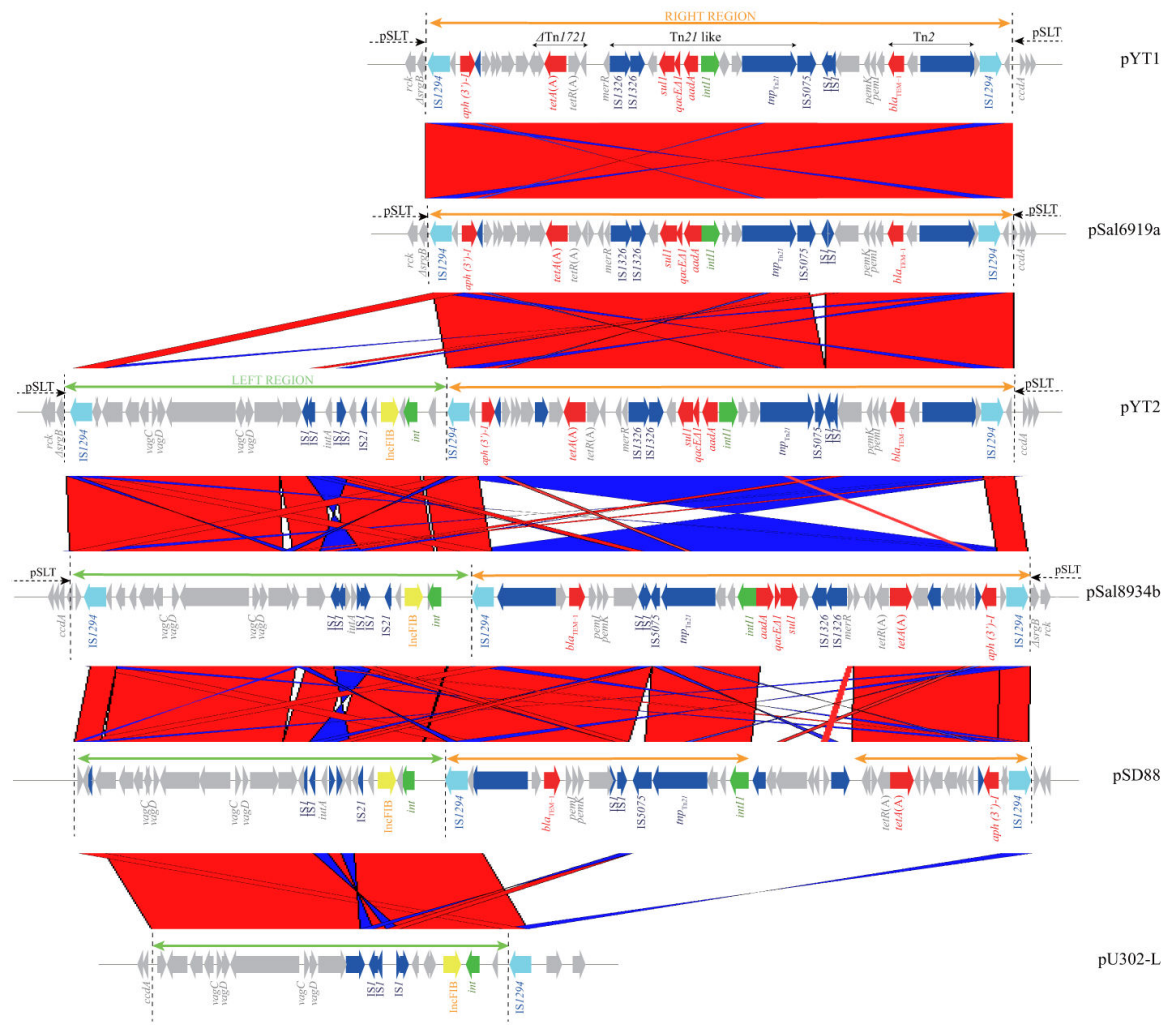
Schematic representation of the inserted segment in pYT1 (GenBank accession number, AB576781) and pYT2 (AB605179) compared with the respective related regions of pSal6919a (JF274991), pSal8934b (JF274992), pSD88 (JF267652), and pU302-L (NC 006816). Genes encoding antibiotic resistance (red), IS*1294* transposase functions (right blue), other transposase functions (navy), replication function (yellow), integrase functions (green), and other functions (gray) are shown. The black double-arrowheads above the diagram indicate mobile elements. The green and orange double-arrowheads above the schemes indicate the left and right region of pYT1 and pYT2, respectively. The regions similar to these are also indicated by the green and orange double-arrowheads above the diagrams. Pair-wise alignment of sequences was performed using a BLASTN similarity search and visualized using the ACT program. The red and blue bars between diagrams represent individual nucleotide matches in the forward or reverse directions, respectively.

Nucleotide sequencing showed that pYT1 and pYT2 are 112,670-base pairs (bp) and 132,842-bp long, respectively, and closely related to pSLT, a virulence-associated plasmid essential for systematic invasiveness of *S*. Typhimurium in mice [12]. The pYT1 and pYT2 sequences consist of segments derived from pSLT DNA and single large inserts (32,495 bp in pYT1 and 52,666 bp in pYT2). The inserted DNA in both plasmids consists of a region bracketed by two copies of *IS1294* in opposite orientations and some flanking sequences. In pYT1 and pYT2, the inserted DNA is located between the 3′ end of the truncated *srgB* and the noncoding region upstream of *ccdA* (Figure 1). The deleted segment of pSLT, including a part of *srgB*, *srgA*, the *pef* operon (*pefIDCAB*), and IncFIB/*repA2*, which is one of the two replicons of pSLT, is replaced by the acquired DNA. The pYT1 sequence is mostly identical to that of pYT2, except that it lacks the left region (21,015 bp; position 3,125–24,139 in pYT2) flanked by two copies of IS*1294*, which is nearly identical to the segments of plasmids as follows: *S*. Typhimurium pU302L (GenBank accession number, NC 006816) [13] and S. Dublin pSD88 (GenBank accession number, JF267652) [14] (Figure 1). Neither pU302-L nor pSD88 are pSLT-like. The left region in pYT2 includes genes encoding plasmid maintenance (*vagC* and *vagD*), the IncFIB replicon, and the iron acquisition-associated virulence gene (*iutA*) flanked by two copies of IS*1* elements. The IncFIB replicon of pYT2, which is allele B1 by replicon sequence typing [15], differs from that in pSLT (allele B17), indicating that it did not originate from pSLT. Because the left region is flanked by two copies of IS*1294* in the same orientation, it could be gained or lost by recombination in IS*1294*.

The right region common to pYT1 (32,495 bp; position 3,129–35,623) and pYT2 (31,651 bp; positions 24,140–55,790) contains entire resistance genes (Figure 1). Both harbor resistance clusters comprising a Tn*21*-like transposon containing an In2-like class 1 integron, which carries the resistance genes *aadA1*, *qacEΔ1*, and *sul1* (resistance to streptomycin-spectinomycin, ammonium antiseptics, and sulfonamides, respectively). In2 itself has both IS*1326* and IS*1353* [16], but only IS*1326* is present in these plasmids. The Tn*2* transposon carrying *bla*_TEM-1_ (ampicillin resistance) is located upstream of the right-terminal IS*1294* in both plasmids and is truncated by IS*1294*. The IS*5075* element interrupting the 38-bp terminated inverted repeat of Tn*21* in pYT1 and pYT2, is intact in pYT1, but truncated by insertion of IS*1* in pYT2.The region between the inverted repeat of Tn21-like and Tn*2*, which contains the *pemI* and *pemK* toxin-antitoxin genes, is 100% identical to a segment of IncFII plasmid NR1 (GenBank accession number AP000342) with the same boundary with IS*1*. A gene encoding kanamycin resistance, *aph* (*3’*)*-I*, is located upstream of the left-terminal IS*1294* in the right region. The *tetA*(A) and *tetR*(A) genes, part of Tn*1721*, are located between *aph*(*3’*)*-I* and the Tn*21-*like transposon. The plasmid pSD88 contains only part of the right region (Figure 1). Both plasmids contain a 648-bp stretch downstream of the right end of IS*1294* that is 100% identical to a segment of pU302-L and differs by one nucleotide from the corresponding sequence in pSD88. The plasmid pYT2 contains a 180-bp sequence downstream of the left end of the IS*1294* element (position 3125–3304) that is not present in pYT1.

To investigate whether pYT1 and pYT2 were self-transferable, conjugation experiments were performed using *E. coli* C600 Nal^r^ as the recipient; however, no transconjugants were obtained. The plasmid pSLT is self-transferrable [17], and pYT1 and pYT2 have all *tra* genes in common with pSLT. However, there is a deletion of 81-bp in *traD* of pYT1 and pYT2 compared with that of pSLT. Thus, the sequence of *traD* in the former two plasmids matches that of the non-self transferable plasmid pSLT_SL1344 (GenBank accession number HE654724) [17], and these changes may result in lack of conjugation.

Antibiotic resistance-encoding derivatives of pSLT, such as pUO-StVR2 [18], pSTMDT12 [10], and pSLT-BT[19] consist of pSLT with different insertions as found in pYT1 and pYT2. Isolates carrying pUO-StVR2 were first detected in Spain in 1993 and subsequently disseminated widely in Europe [10]. The plasmid pUO-StVR2 carries a 46,066-bp segment containing the *vagCD* region and drug-resistance regions. The insertion site of foreign DNA in pUO-StVR2 is almost the same as those of pYT1 and pYT2. Different IS*26*-mediated composite transposons carrying different resistance genes are inserted in different places in the plasmid backbone in pSTMDT12 and pSLT-BT. Similarly, IS*1294* elements were identified in the inserted fragment in pYT1 and pYT2, suggesting that multidrug-resistance genes were acquired through IS*1294*-related integration.

A single copy of the IS*1294* element can promote transposition of sequences adjacent to one end of the element by a rolling circle mechanism [20,21]. Sequence analysis suggests that pSD88 and pU302-L may be the source of the inserted regions in pYT1 and pYT2. Further, both pSD88 and pU302-L harbor IS*1294* elements (Figure 1). Therefore, pYT1 and pYT2 may have arisen from the virulence plasmid pSLT by acquiring IS*1294* and adjacent sequences of pSD88, and/or pU302-L or those of related plasmids through the integration of IS*1294*, followed by inversion and recombination.

In addition to antibiotic resistance genes, pYT2 contains the putative virulence gene *iutA*. Therefore, the virulence plasmid pSLT may act as a gene-exchange platform, facilitating the sampling of genes collected from other bacteria in the environment as suggested by Kingsley et al [19]. The presence of resistance and virulence determinants on the same plasmid allows co-selection of both properties by antimicrobial agents; more virulent and antibiotic-resistant bacteria may subsequently arise.

Interestingly, BLAST analysis reveals that pYT1 is 99% identical to pSal6919a (GenBank accession number, JF274991) (3-bp difference). There are the regions in pYT2 that are inverted compared with pSal8934b (GenBank accession number, JF274992). When this is taken into account, the two plasmids share 99% sequence identity (218-bp difference) (Figure 1). Both pSal6919a and pSal8934b consist of sequences derived from pSLT and an inserted region flanked by IS*1294* that are almost identical to those of pYT1 and pYT2, respectively. Their structural similarity indicates that pYT1, pYT2, pSal6919a, and pSal8934b may have been generated by recombination between IS*1294* elements, inverting the regions between them. These results suggest that similar multidrug-resistant plasmids may have disseminated elsewhere as well.

TST207, a PFGE cluster VII isolate resistant to cefotaxime, harbors a *bla*_CMY-2_-carrying plasmid designated pYT3 and carries the virulence-resistance plasmid pYT4, which is almost identical to pYT1 (data not shown). The pYT3 sequence predicts 148 coding sequences totaling 121,723 bp, including *bla*_CMY-2_ and five antimicrobial-resistance genes in two resistance regions (Figure S1). The *floR* region includes *floR* (chloramphenicol/florfenicol resistance), *tetA* (A) (tetracycline resistance), *strA*, *strB* (streptomycin resistance), and *sul2* (sulfonamide resistance). The “*bla*_CMY-2_” region comprises a complete transposable element IS*Ecp1*-*bla*_CMY-2_-*blc1*-*sugE1* array. Most *tra* genes required for conjugation and plasmid transfer are present (Figure S1); however pYT3 lacks *trhF, traW*, and *traU*, which are present in other *bla*_CMY-2_-carrying plasmids described below. Therefore, pYT3 was not transferred to *E. coli* C600 Nal^r^ by conjugation.

Much of pYT3 shares sequence similarity with the sequences as follows: *bla*_CMY-2_-positive IncA/C plasmids pAR060302 (166,530 bp) of *E. coli* and pSN254 (176,473 bp) of *S. enterica* serovar Newport, and the 125,122-bp genomic island GI-VII-6, possibly derived from pAR060302. The latter island contains antibiotic-resistance genes, including *bla*_CMY-2_, which is located in the chromosome of cefazolin-resistant *S*. Typhimurium PFGE cluster VII [11] (Figure S1). Alignment of pYT3 and pAR060302 shows that a large portion of pYT3 (77.2%) is 99% identical (7-bp difference) to part of pAR060302 (Figure S1). Interestingly, a 24,207-bp sequence in pYT3 (92,306 bp to 116,512 bp) contains the IncFIB replication gene (allele B1), which is flanked by two copies of IS*1294*. This segment is similar to the 21,015-bp left region in pYT2, which also contains an IncFIB replication gene flanked by two copies of IS*1294* (Figure 1). Although pYT3 shares sequence identity with the IncA/C plasmids, no replication gene other than IncFIB was identified. Therefore, pYT3 may have been generated by IS*1294*-mediated recombination between segments in pAR060302 and the region containing IncFIB replicon in the pYT2-like plasmid found in isolates of PFGE cluster VII. Further, the IncA/C replication region might have been replaced by the IncFIB replication region in pYT3. Taken together, these results indicate that pYT3 and the genomic island of GI-VII-6 in an emerging multidrug-resistant clone share a common ancestry with *E. coli* IncA/C plasmid pAR060302.

Southern hybridization analysis failed to detect *bla*_CMY-2_ in the chromosome of TST207, the isolate carrying pYT3 (data not shown). Therefore, extra-chromosomal copies of *bla*_CMY-2_ in pYT3 seem to increase the level of β-lactam resistance in TST207, which is resistant to cefotaxime. Recently, isolates of PFGE cluster VII resistant to expanded-spectrum cephalosporins mediated by *bla*_CMY-2_ in an IncA/C plasmid were isolated in Japan [22]. Because pYT3 lacks an IncA/C-specific replicon initiation gene (*repA*), pYT3 differs from these IncA/C plasmids.

The results of the present study support a plausible mechanism of evolution and dissemination of multidrug resistance in the emerging multidrug-resistant clone of *S*. Typhimurium PFGE cluster VII.

## Supporting Information

Figure S1 **Circular representation of pYT3 from S.Typhimurium TST207 belonging to PFGE cluster VII.** The inner-most and second inner-most circular diagrams show the positions of coding sequences transcribed in the counterclockwise and clockwise directions, respectively. Genes encoding antibiotic resistance (red), IS*1294* transposase functions (blue), replication function (yellow), and other genes (gray) are shown. The third circle shows the nucleotide positions. The outer circles indicate regions homologous to pAR060302 (red), GI-VII-6 (orange), and pYT2 (green).
(EPS)
